# Current Landscape of Genome-Wide Association Studies in Acute Myeloid Leukemia: A Review

**DOI:** 10.3390/cancers15143583

**Published:** 2023-07-12

**Authors:** Richard J. Marrero, Jatinder K. Lamba

**Affiliations:** 1 Department of Pharmacotherapy and Translational Research, College of Pharmacy, University of Florida, Gainesville, FL 32610, USA; 2 University of Florida Health Cancer Center, University of Florida, Gainesville, FL 32610, USA; 3 Center for Pharmacogenomics and Precision Medicine, College of Pharmacy, University of Florida, Gainesville, FL 32610, USA

**Keywords:** acute myeloid leukemia, genomics, pharmacogenetics, pharmacogenomics, SNP

## Abstract

**Simple Summary:**

Acute myeloid leukemia is a rare blood cancer that develops from the clonal expansion of malignant myeloid precursor cells located in the bone marrow. Despite a relatively low incidence in the general population as compared to other cancers, it is one of the most common types of hematological cancers in adults. In recent years, various genome-wide association studies have been conducted to examine how genetic variation impacts disease risk and clinical outcomes in patients diagnosed with acute leukemias. Overall, in the field of cancer research, numerous genome-wide studies have been performed, however, there is a lack of studies specifically focused on patients diagnosed with acute myeloid leukemia. This review provides a summary of the recent genome-wide studies conducted in acute myeloid leukemia, focusing on challenges and limitations associated with research of this heterogenous and rare disease.

**Abstract:**

Acute myeloid leukemia (AML) is a clonal hematopoietic disease that arises from chromosomal and genetic aberrations in myeloid precursor cells. AML is one of the most common types of acute leukemia in adults; however, it is relatively rare overall, comprising about 1% of all cancers. In the last decade or so, numerous genome-wide association studies (GWAS) have been conducted to screen between hundreds of thousands and millions of variants across many human genomes to discover genetic polymorphisms associated with a particular disease or phenotype. In oncology, GWAS has been performed in almost every commonly occurring cancer. Despite the increasing number of studies published regarding other malignancies, there is a paucity of GWAS studies for AML. In this review article, we will summarize the current status of GWAS in AML.

## 1. Introduction

Acute myeloid leukemia (AML) is a heterogenous hematologic malignancy characterized by the unregulated clonal expansion of abnormal myeloid progenitor cells in bone marrow. Immature leukemic cells accumulate in the bone marrow and blood, eventually infiltrating other organs, which disrupt production of normal blood cells. As the disease progresses, weakness, infection, bleeding and a myriad of other complications occur in the absence of treatment. AML can be categorized as de novo or secondary AML, with the latter subtype attributed to progression of other diseases or being a result of treatment with radiation or cytotoxic agents (known as therapy-related AML). Even with recent advancements in medical technology, treatment of patients diagnosed with AML remains challenging, with high rates of relapse and refractory disease contributing to dismal outcomes. Optimizing chemotherapy regimens to reduce adverse drug reactions and improve tolerability while attaining maximum therapeutic effect remains an unmet need. 

Although it is the most common category of acute leukemia in adults, AML is considered a rare disease, having an approximate incidence rate in the United States of 4.1 per 100,000 men and women per year [1]. Diagnosis of AML is most frequent in persons aged 65–74, with the median age at diagnosis being approximately 68 years old [1]. Death rates are highest among persons aged 65 years and older, with an age-adjusted overall death rate in the United States (during the period 2016–2020) of 2.7 per 100,000 men and women [1]. Despite newer targeted therapies and advances in supportive care regimens, the 5-year relative overall survival rate remains a modest 30.5% [1]. Aside from chromosomal alterations, epidemiological studies have associated various environmental factors, such as exposure to ionizing radiation, chemical or biological compounds such as benzenes or other solvents, as well as tobacco use, with AML [2,3,4].

The numerous chromosomal and genetic abnormalities make AML challenging to treat. Current treatment approaches utilize an integrated approach whereby phenotypic and genetic data are used to stratify patients into risk groups at diagnosis and during treatment. Although risk stratification has improved prognostication, a more thorough understanding of AML will likely contribute to more personalized approaches to treatment, with the overarching goal being improving survival outcomes and maintaining a durable remission. Consistent with the polygenic nature of complex diseases, hundreds of genes with recurring mutations that detrimentally affect hematopoiesis have been identified; however, it has been shown that a small number of recurrent mutations in driver genes are attributed to those portions of the genome that act as coding regions [5,6,7]. With the advent of innovative sequencing methods, focus is shifting toward the patient’s genomic profile, with this focus being particularly salient for the more than 50% of patients who present a normal karyotype at diagnosis [6].

## 2. Evolution of Genetic Studies in AML

Initially, early studies focused on heritability within families that contained clusters of AML, which identified a few highly penetrant germline mutations that confer higher risk [8,9,10]. More recently, there has been increasing recognition of the fact that germline mutations may be associated with increased risk of development of AML and/or myelodysplastic syndromes (MDS), and identifying these subsets of patients is important in improving outcomes [11,12]. As a result, in 2016, the World Health Organization (WHO) updated their classifications for patients with acute leukemias, MDS, or myeloproliferative neoplasms with an underlying germline mutation to include “myeloid neoplasms with germline predisposition”, which was subsequently incorporated into the National Comprehensive Cancer Network’s (NCCN) Clinical Practice Guidelines [13,14]. Use of direct high-depth sequencing techniques to assist in differentiating de novo AML from other myeloid diseases, as well as inherited forms or those relating to clonal hematopoiesis, is essential [15]. Molecular recognition of germline predisposition includes the following genetic susceptibility factors: RUNX family transcription factor 1 (RUNX1), CCAAT enhancer-binding protein α (CEBPA) and GATA binding protein 2 (GATA2); all of these proteins are regulatory transcription factors, impact hematopoiesis and have been associated with a younger age of AML onset [16,17].

The lack of definitive findings from family-based linkage studies and other epidemiological reports underscore the polygenic nature of AML. Thus, GWAS-based approaches can facilitate the discovery of novel genetic variants in genes of biological relevance to AML. The premise of GWAS is based on the “common disease—common variation” hypothesis and uses a case–control study design [18]. The effects of single nucleotide polymorphisms (SNPs) on disease risk, clinical outcome, or other disease-related traits of interest can be explored using a GWAS-based approach. Generally speaking, GWAS utilize whole-genome genotyping using microarray-based technologies either with or without unobserved genotype imputation. Numerous GWAS have identified coding and non-coding variants across numerous complex diseases, the results of which have been used to inform disease risk or elucidate underlying biological mechanisms that can assist in identifying patients associated with a particular clinical outcome. As of July 2022, the National Human Genome Research Institute (NHGRI) and the European Bioinformatics Institute (EBI) GWAS Catalog contains approximately 400,000 SNP–trait associations derived from >45,000 GWAS studies in approximately 6000 published articles [19]. Furthermore, large cohorts with information about a wide range of diseases and traits, such as those contained in United Kingdom (UK) Biobank, have led to an increase in GWAS studies [20]. In this review, we summarize existing GWAS studies in AML and present the challenges and limitations associated with conducting research into this complex and rare disease.

## 3. Literature Search Strategy

An extensive literature search of the electronic databases PubMed and EMBASE was conducted to identify peer-reviewed scientific studies (published on or before 6 January 2023) that reported results from GWAS on AML disease risk and/or clinical outcomes using the search terms “acute myeloid leukemia”, “AML”, “genetic studies”, “genome wide association studies”, “GWAS” and “gene polymorphisms”. The searches were limited to studies of humans and written in the English language. In addition to studies reporting GWAS, we included one study that conducted a haplotype-based association analysis in AML, as well as another study that tested selected SNPs from a published GWAS.

## 4. Results

Using computer database retrieval, 333 articles were identified from PUBMED and EMBASE, of which 186 duplicate studies were removed. After following the exclusion criteria, 22 records were retrieved for further examination and an additional 15 articles were removed because results were not reported solely in relation to AML-diagnosed patients. Therefore, seven studies met the criteria for inclusion in this review (Table 1).

### Summary of GWAS Studies

Study 1 (Knight et al., 2009) [21]: Given that therapy-related acute myeloid leukemia (t-AML) and myelodysplastic syndrome (t-MDS), which are collectively referred to as t-AML, are increasingly common in patients previously treated with cytotoxic drugs, this study reasoned that larger effect sizes would be more readily identifiable in t-AML than in de novo AML or MDS. The study design included discovery (*n* = 80, 62 with t-AML and 18 with t-MDS) and validation cohorts (*n* = 70, 33 with t-AML and 37 with t-MDS) [21]. The control dataset included 150 healthy individuals with no history of cancer or cytotoxic therapy. Genotype information was generated via Affymetrix microarray for the discovery and control cohorts and pyrosequencing for the validation cohort. Of note, all genomic DNA from cases were obtained at the time of diagnosis and prior to administration of cytotoxic chemotherapy drugs for the treatment of t-AML. Though none of the SNPs passed the genome-wide significance level, 15 SNPs demonstrated differential abundance in cases vs. controls of *p* < 0.001. Of these cases, for five SNPs, minor allele was associated with significant risk of t-AML and for 10 SNPs, major allele was associated with significant risk of t-AML. Of note, three SNPs (rs719293, rs2375990, and rs2133508) were completely absent in cases but occurred with a frequency 6–8% in controls. These 15 SNPs were genotyped in independent validation cohorts (70 cases and 95 controls), and after exclusion of five SNPs due to linkage disequilibrium (LD) or minor allele frequency (<0.1 in cases and control), 10 SNPs were further analyzed. Though none of SNPs were validated at the pre-defined significance thresholds, two SNPs (rs1394384 and rs1381392) showed a trend towards significance. Comparing SNPs within both discovery and validation cohorts, six SNPs (rs953509, rs1394384, rs556831, rs1381392, rs374284 and rs1335546) were significant at *p* < 0.05 in both cohorts.

Given prior exposure to alkylating agents has been associated with acquired lesions in either or both chromosome 5 and 7, the authors conducted a subset analysis in patients from both cohorts who had these genetic aberrations. In this homogenous subset, three SNPs, namely rs1394384, rs1381392 and rs1199098, were validated as potential markers associated with t-AML. Of these SNPs, rs1394384 was found in the intron of the Acid Sensing Ion Channel Subunit 2 (*ASIC2*) gene, which encodes an amiloride-sensitive cation channel that belongs to the DEG/ENaC superfamily and was protective of risk to t-AML. SNP rs1199098 was located in ~2 KB upstream of the inositol–polyphosphate multikinase (*IPMK*) gene, and the authors concluded the relevance of *IPMK* due to its interactions with AKT kinase and Wnt/beta-catenin signaling [28]. *IPMK* has also been implicated in transcriptional regulation and inositide metabolism in the nucleus and is, thus, a regulator of nuclear signaling pathways [29,30]. Moreover, it has also been shown to impact autophagy induction by interacting with AMPK/ULK1 signaling [31,32]. The third SNP, i.e., rs1381392, was present in a gene dessert region and, thus, its functional relevance is unclear; however, this region has been shown to have recurrent deletions in lung cancers [33]. The authors did not perform an in-depth functional analysis and concluded that further research is needed to determine the contribution of these variants to t-AML risk in larger cohorts.

Study 2 (Lv et al., 2017) [23]: A genome-wide haplotype association analysis (GWHAS) was performed in this study based on previous research that demonstrated that multi-marker haplotype analysis may have greater capacity to detect correlations with disease. The subject study was restricted to patients with core-binding factor AML [23] derived from data downloaded from the Gene Expression Omnibus (GSE32462). Historically, CBF-AML, which represents t(8:21) translocation and inv 16, accounted for ~15% of AML and was associated with a favorable prognosis [34]. Genomic DNA obtained at remission from pediatric and adult CBF-AML patients (*n* = 175, GSE32462) was genotyped using the Affymetrix SNP6.0 Array. The control dataset consisted of non-AML patients derived from the International HapMap Project (*n* = 218) [35]. Approximately 700,000 autosomal SNPs common to both AML cases and healthy controls were used for haplotype analysis. Out of 118,057 haplotype blocks identified, 519,865 haplotypes were tested, and 1754 showed significant correlation with AML at *p* < 1 × 10^−5^ and mapped to 591 genes. Using a novel candidate gene approach to prioritize haplotypes [36], four high-risk AML genes with significant association were ranked: *RUNX1*, *JAK1*, *PDGFRA* and *FGFR2*. The Runt-related transcription factor 1 (*RUNX1*) gene is well-characterized in CBF-AML and other hematologic malignancies, and it is known to impact hematopoiesis [37]. The Janus kinase 1 (*JAK1*) gene is a member of the JAK/STAT signaling pathway and involved in the regulation of various cellular processes, including cell growth and differentiation, as well as immune responses. It has been implicated in the pathogenesis of several types of leukemia, including acute lymphoblastic leukemia (ALL) and AML [38]. The platelet-derived growth factor receptor alpha (*PDGFRA*) gene is more commonly associated with other types of AML, such as the subset of AML with eosinophilia and abnormalities of chromosome 5 or 7 [39]. Although the fibroblast growth factor receptor 2 (*FGFR2*) gene is not typically altered in CBF-AML, mutations in *FGFR2* have been associated with many other malignancies [40]. Moreover, translocation at the *FGFR2* locus have been previously reported in multiple myeloma and myeloproliferative disorders [41].

The presence of *RUNX1*, which is the primary susceptibility gene associated with CBF-AML in this study, is not surprising, given that the genotype data are enriched in patients with CBF-AML. However, its association with the other prioritized genes is intriguing. Due to the molecular heterogeneity in AML, genetic abnormalities can vary between individual cases of leukemia, and there may be rare cases where *JAK1*, *PDGFRA* and *FGFR2* abnormalities are present in CBF-AML. Nonetheless, the presence of mutations or aberrations in these genes is not a typical feature of CBF-AML. Of note, no other studies used GWHAS in AML to investigate the way in which genetic variation impacts risk of AML.

Study 3 (Bargal et al., 2018) [24]: Resistance to cytarabine and high relapse rates remain a major barrier to successful treatment in AML patients. As a result, Bargal et al. conducted a GWAS to investigate SNPs associated with the chemosensitivity of AML patients’ diagnostic leukemic cells to cytarabine [24]. The study included pediatric AML patients who were participants in the multi-center St. Jude AML02 study [NCT00136084], and specimens of their leukemic cells were tested for cytarabine chemosensitivity. Patients with high-risk group features were more frequent among subgroups whose cells were resistant to cytarabine. Due to the small sample size, the study population was limited to 50 genetically Caucasian patients; thus, none of the variants reached genome-wide significance. Using an a priori *p* < 1 × 10^−4^ suggestive significance threshold, the primary SNP, i.e., rs721947, which was located on chromosome 12, was most strongly associated with cytarabine resistance. It is located downstream of NEDD1 Gamma-Tubulin Ring Complex Targeting Factor (*NEDD1*) gene and situated within a long intergenic non-coding RNA region. Another SNP, i.e., rs1376041, is located in an area close to the exon/intron junction of the Adhesion G Protein-Coupled Receptor G1 (*ADGRG1*) gene (also referred to as *GPR56*). The minor allele A for rs1376041 was associated with resistance to cytarabine. *ADGRG1* has been shown to participate in cancer-cell proliferation and associated with inferior results in AML [42,43]. Another SNP below suggestive significance, i.e., rs75400242, is located upstream of the Insulin-Like Growth Factor 1 Receptor (*IGF1R*) gene, which is expressed in human leukemia cells and was previously shown to be associated with AML [44]. *IGF1R* is one of the phosphorylated receptor tyrosine kinases present in AML cases, and it has been linked to mutant RAS-positive AML, having the potential therapeutic benefits of dual suppression of IGF1R and RAS signaling [44]. To enhance the validity of the results, Bargal et al. conducted an expression quantitative trait loci (eQTL) analysis to examine the correlation between SNPs identified via GWAS analysis and the expression of respective genes in the same samples, in addition to associations between gene expression and cytarabine sensitivity. The authors sought to evaluate primary candidate signals using a three-point criteria: (i) SNP vs. cytarabine chemosensitivity of primary leukemic cells; (ii) SNP vs. gene-expression levels; and (iii) gene-expression vs. chemosensitivity. Two genes, i.e., *ADGRG1* and *IGF1R*, satisfied all three criterion and were chosen for in vitro mechanistic studies. Small inhibitory RNA (si-RNA)-mediated knockdown of *ADGRG1* and *IGF1R* in AML cell lines confirmed the observations that primary AML cells with knock down resulted in increased apoptosis and, thus, sensitivity to cytarabine. This study utilized the most comprehensive collection of in vitro cytarabine chemosensitivity data from pediatric AML patients. However, the small sample size warrants additional research to confirm these results, as well as in-depth functional studies to establish the underlying mechanism that causes cytarabine resistance.

Study 4 (Walker et al., 2019) [25]: To evaluate the risk of AML, Walker et al. conducted a GWAS using three patient cohorts that consisted of 1533 adults of European descent diagnosed with de novo AML and 3969 non-AML controls [25]. Following imputation, approximately 16 million autosomal variants were tested in 1183 AML cases and 2369 controls of European ancestry. The discovery phase included two independent case–control cohorts of AML patients from the United States. The results of the association tests determined via meta-analysis of the combined discovery cohort sample sets identified 11 polymorphisms at five loci that were associated with AML at a suggestive significance threshold of *p* < 10 × 10^−6^ (none of the variants achieved genome-wide significance). To validate the markers with the lowest *p*-values, an independent cohort of German cases and controls was used. Chromosomal loci 19q13, 19p13 and 13q22 were selected for validation due to the presence of SNPs with lowest overall *p*-values and representation of two or more variants below suggestive significance levels. The two SNPs located on chromosome 19 were validated using a third independent cohort, and SNP rs57706619 and the 19q13 SNP rs75797233 showed significant association with AML. Next, a meta-analysis combining all the cohorts confirmed the association between rs75797233 (*p* = 4.15 × 10^−8^; Odds ratio: 2.28) and AML risk.

SNP rs75797233 is located 13KB from BRD4 Interacting Chromatin Remodeling Complex Associated Protein (*BICRA*) gene. *BIRCA* is a candidate tumor suppressor gene for glioma and has been implicated in transcriptional regulation in colorectal cancer [45,46]. The Genotype-Tissue Expression (GTEx) project database showed that the rs75797233-A allele is associated with higher *BICRA* expression in whole blood. Furthermore, chromatin immunoprecipitation (ChIP) sequencing data confirmed the presence of a GATA2 transcription factor binding site in this region, as well as that the presence of the risk allele repressed a critical residue at the GATA2 binding site. Using quantitative reverse transcription polymerase chain reaction (PCR) and ChIP quantitative PCR, it was shown that cell lines heterozygous for the rs75797233 risk allele had significantly reduced *BICRA* expression and reduced GATA2 binding to the locus. These results suggest that rs75797233 may be associated with AML risk through regulating *BICRA* expression through GATA2; however, in-depth mechanistic studies are needed to establish the role of *BICRA* expression in the development of AML. Although this past study was one of the largest AML disease risk GWAS conducted, the genome-wide association in *BICRA* was not reported in other independent cohorts of AML patients [26,27]. This issue may be due to the low minor allele frequency of rs75797233 (0.02); thus, other GWAS studies may not be powered to detect the lower frequency of the effect allele in independent cohorts. The second SNP on chromosome 19 included rs57706619, which is an intronic SNP located in the BetaGal Beta-1,3-N-Acetylglucosaminyltransferase 3 (*B3GNT3*) gene. The third SNP, i.e., rs2039647, was only significant in one of the three cohorts and mapped to chromosome 13q22. This haplotype block included the long non-coding RNA LINC00402 and KLF Transcription Factor 12 (*KLF12*) gene, which encodes a protein that is a member of the Krüppel-like zinc finger protein family and has been broadly associated with various other cancers types.

Study 5 (Lin et al., 2021) [26]: This manuscript reported results from three independent GWAS that examined 3041AML cases and 6760 controls of European ancestry using genotype data [26]. Publicly available genotyping data used in this study have been deposited in the NCBI Gene Expression Omnibus under the following accession numbers: GSE20672, GSE32462, GSE34542, GSE46745 and GSE46951. Imputation was used to estimate missing genotypes for over 7 million variants. The association test statistics were pooled for 6,694,056 SNPs that were common to all three GWAS, followed by a meta-analysis that was conducted to identify variants associated with AML. Findings from the meta-analysis were replicated in a fourth cohort of patients, with similar effect sizes of associations across all four studies. Given the heterogeneity of AML, subgroup analysis was performed, with emphasis placed on the largest group, which consisted of patients with cytogenetically normal AML (CN-AML, *n* = 822). Within the discovery cohort, genome-wide significant (*p* < 5 × 10^−8^) susceptibility loci were observed in CN-AML and included SNP rs3997854 located in Major Histocompatibility Complex, Class II, DQ Alpha 2 (*HLA-DQA2*) gene (Chr 6p21.32) and rs75391980 (Chr 4q22.3). Of note, a few other SNPs reached significance at a suggestive threshold of (*p* < 10^−6^), including rs4674579, rs2621279, rs13183143, rs11481 and rs6077414.

Validation of a fourth independent cohort (*n* = 977 cases, of which 465 were CN-AML, and *n* = 3728 controls) and meta-analysis confirmed the presence of rs4930561 in Lysine Methyltransferase 5B (*KMT5B*) gene on Chr 11q13.2 in all-AMLregardless of subtypes and rs3916765 in *HLA-DQ2* (Chr 6p21.32) in CN-AML. Further meta-analysis of SNPs common to all four cohorts identified rs10789158 in Cache Domain Containing 1 (*CACHD1*) at Chr 1p31.1 risk locus in a consistent direction for all-AML and rs17773014 in the Aldo-Keto Reductase Family 1 Member B (*AKR1B1*) gene at Chr 7q33 for CN-AML. These results remained consistent when analysis was performed to correct any underlying effects of population ancestry by re-examining cases and controls of British and German origin present in the four GWAS cohorts. An attempt to test these variants in a cohort of 767 AML patients from the UK, Germany and Hungary did not find any association between the variants and relapse-free or overall survival in all-AML or CN-AML patients (*n* = 369). SNP rs4930561 was reported to be significantly associated with risk of AML, regardless of subtype. The variant is located in the *KMT5B* gene on chromosome 11q13.2 and a member of the histone–lysine methyltransferases (KMTs) family, which are essential in many tightly regulated cellular processes. Dysregulation of KMTs has been associated with many cancers [47,48]. Using the eQTLGen consortium [49], the authors showed this SNP to be associated with expression of 12 of the 47 genes mapped in 500KB of the SNP on Chr 11q13.2; however, *KMT5B* expression did not show significant association with the lead SNP.

The second genome-wide significant susceptibility locus for CN-AML was identified at Chr 6q21.32 in *HLA-DQA2* gene. This region has been linked to an increased risk of numerous human cancers, including various hematological malignancies [50,51,52]. Evaluation of HLA alleles in 5225 individuals of European ancestry showed the HLA DQB1*03:02 and HLA-DQA1*03:01 alleles to be significantly less common in AML cases than in controls, thus implying that abnormal immune systems are associated with risk of CN-AML. Of note, the previously reported AML risk variant in the *BICRA* gene (rs75797233) was not found to have a significant association with AML in this study. One of the reasons for this finding might be its rarity and the fact that it was only imputed to a high enough quality in three GWAS studies. These data bolster the existing evidence of genetic and biological diversity in AML and highlight the need for collaborative studies to increase statistical accuracy and aid in the discovery of genetic risk loci that are specific to AML sub-types.

Study 6 (Wang et al., 2021) [27]: This large-scale GWAS in patients diagnosed with AML and myelodysplastic syndrome (MDS) in an unrelated donor bone marrow transplant population was conducted with the rationale that inherited genetic variants may overlap and contribute to the development of both AML and MDS [27]. Patients from DISCOVeRY-BMT cohorts (2309 cases and 2814 controls) from the Center for International Blood and Marrow Transplant Research (CIBMTR) [53,54] were evaluated, and SNP rs12203592 located at Chr 6p25.3 within the interferon regulatory factor 4 (*IRF4*) gene was associated with an increased risk of de novo AML, de novo MDS and therapy-related MDS, but not therapy-related AML. *IRF4* is a transcription factor involved in T and B lymphocyte differentiation. SNP rs12203592 is implicated in *IRF4* regulation via interactions with the chromatin loop [55]. Though it was the largest genome-wide investigation of AML and MDS susceptibility loci, some associated limitations include variations in exposure to therapy for therapy-related MDS and AML, the heterogeneity of cytogenetic risk groups, and the predominantly non-Hispanic European Americans ethnic backgrounds of the patients studied.

Study 7 (Cao et al., 2016) [22]: Though not a standalone GWAS, a study by Cao et al. tested 16 SNPs that were previously identified as susceptibility loci in two prior GWAS conducted in AML patients of European ancestry [22,56,57]. The goal was to test if these markers could be replicated in AML patients of Han Chinese ancestry. A case–control study with 545 newly diagnosed AML cases and 1034 controls was conducted, and regression models were adjusted for age and gender. Three SNPs, i.e., rs2191566, in zinc finger protein 230 (*ZNF230)*; rs9290663, in potassium calcium-activated channel subfamily M regulatory beta subunit 2 (*KCNMB2*); and rs11155133, in non-coding RNA gene (LOC102723724), were associated with a higher risk of developing AML, whereas rs10873876 in ST6 N-acetylgalactosaminide alpha-2,6-sialyltransferase 3 (*ST6GALNAC3*) was associated with s lower risk of developing AML.

Among these primary SNPs, rs2191566 in *ZNF360* was associated with a higher risk of M2-AML in cases with AML1/ETO fusion but not in cases without fusion, suggesting a subtype-specific impact of the SNP. *ST6GALNAC* SNP showed an inconsistent association relative to previous report; this lack of association was speculated to be due to the different study populations: ALL vs. AML compared to European vs. Chinese patients. Further investigation of the SNP in LOC102723724 using in silico databases predicted that A to G base-change might affect the binding of C/EBP; however, current evidence of this underlying mechanism warrants further research. While not an independent GWAS in AML, the objective of this replication study was to replicate findings from two previous GWAS studies on acute leukemias. However, ALL vs. AML differences, sample size limitations, and potential gene–environment interactions limited efforts at validation of the previous findings.

**Figure 1:**
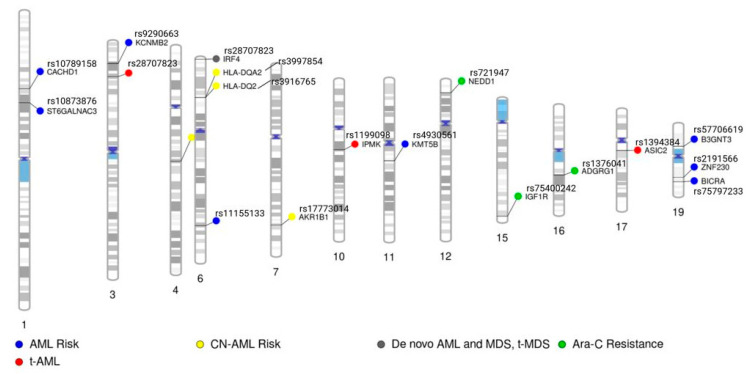
Graphical summary based on chromosomal location of single nucleotide polymorphisms associated with risks or clinical outcomes, as reported in GWAS studies.

Figure 1 shows a summary of GWAS SNPs reported in the studies listed above, with annotation of the gene or nearest gene region in applicable SNPs.

## 5. Future Directions

While GWAS studies have been successful in other complex diseases, there has been a relative lack of such studies in relation to AML. This issue is partly due to challenges and limitations associated with conducting GWAS studies in AML. The rarity of the disease, the heterogeneity of AML subtypes, and the large and well-characterized cohorts required to identify statistically significant associations are some of these challenges. Additionally, AML is a complex disease in which both genetic and environmental factors contribute to disease risk, making it difficult to distinguish the relative contribution of each type of factor.

This article highlights findings from a handful of GWAS studies conducted in relation to AML. Overall, there are several limitations common to the majority of the studies included in this review. Other than one small study performed in pediatric AML patients, all studies were conducted in predominantly adult patient cohorts. Age is associated with distinct genetic and molecular AML characteristics [58]; as such, differences in age between and within the cohorts likely contributes to the variability in clinical factors, such as overall survival, treatment tolerability and response, and other clinically relevant patient characteristics. It is important to note that AML cases in these studies were derived from patients of mostly European ancestry; thus, results may not be generalizable to other populations, underscoring the need for more diverse representative genetic datasets.

Due to the rarity of AML cases, insufficient sample sizes, which reduce statistical power, make it challenging to detect significant associations. Thus, combining diverse AML cohorts or including MDS patients in genetic studies can introduce several challenges and limitations. AML and MDS are heterogeneous diseases with multiple subtypes and varying genetic profiles. Combining patients with different clinical features can introduce confounding factors that may impact the interpretation of genetic associations. Variability in treatment response, survival outcomes, and disease progression can mask or distort the true genetic associations within each disease entity. Furthermore, grouping diverse subtypes together can limit researchers’ ability to identify subtype-specific genetic alterations and their functional consequences. While combining AML with MDS patients certainly increases cohort size, it further expands the heterogeneity and limits the power to detect risk alleles; thus, it would be recommended to conduct separate genetic studies for AML and MDS whenever possible. It is important to carefully consider and address the inherent heterogeneity and potential confounding factors when interpreting and generalizing the results.

Another common feature of these studies has been the focus on effects of SNPs. These factors highlight the need to modify current approaches by performing GWAS studies in addition to establishing cohorts of larger sample size and integrating cases with other omics data, as well as established somatic cytogenetic and genetic lesions. Further integration of top hits to create polygenic risk scores may increase researchers’ ability to estimate the cumulative contribution of AML susceptibility loci to disease risk.

In recent years, there has been growing awareness of the importance of data sharing in scientific research, and several initiatives have been launched to promote and facilitate data sharing in genetics and genomics research. For example, the Global Alliance for Genomics and Health (GA4GH) is an international organization that aims to promote data sharing in genomics research through the development of standardized tools, policies and frameworks [59]. These initiatives can be instrumental to performing genetic association studies for rare diseases, such as AML. However, despite these initiatives, there are challenges associated with access to data, which may include concerns about patients’ privacy and requirements for computational and cloud computing resources. To address the issue of data sharing in AML research, the Beat AML Master Trial created a comprehensive dataset of genomic, clinical and treatment data for adult AML patients [60]. Additionally, the National Cancer Institute launched the Genomic Data Commons (https://gdc.cancer.gov/, accessed on 11 June 2023), which is a platform that allows researchers to access and analyze genomic and clinical data from numerous cancer studies. In summary, data sharing is essential for maximizing the scientific value of research data, and efforts to promote and facilitate data sharing in AML research should be continued and expanded.

## 6. Conclusions

Despite the success of GWAS studies in other complex diseases, there is paucity of such studies in AML, which highlights the unmet need for comprehensive and collaborative initiatives to enhance identification of risk alleles and development of polygenic scores that are associated with AML disease or treatment outcomes, especially in specific cytogenetic subgroups. One major limitation is that the majority of studies have been performed in patients with predominantly European ancestry, thus warranting the creation of future studies that consider patients from other racial/ethnic groups. Overall, genome-wide studies in AML offer opportunities to advance our understanding of the disease, improve patient outcomes through facilitating personalized medicine approaches.

## Figures and Tables

**Table 1:** Significant results of published genome-wide association studies regarding acute myeloid Leukemia.

Reference	Phenotype	SNP(s)	Gene or Nearest Gene Symbol	Gene Information and Reference to Any Known Association with Cancer/Leukemia
[21]	Therapy-related AML	rs1199098	IPMK	Involved in inositol–trisphosphate metabolic process and necroptotic process. Associated with various cancers, such as breast, prostate, osteosarcoma, and papillary thyroid cancer. (PMIDs: 35864548, 37193176, 36632464, 36186479)
		rs1381392	LINC00693	RNA gene of lncRNA class
		rs1394384	ASIC2	Predicted to enable ligand-gated sodium channel activity. Increased expression in colorectal cancer is associated with poor prognosis (PMID: 28927426)
[22]	AML disease risk	rs2191566	ZNF230	Predicted to enable DNA-binding transcription factor activity, as well as RNA polymerase II-specific and RNA polymerase II cis-regulatory region sequence-specific DNA binding activity. Predicted to be involved in regulation of transcription via RNA polymerase II. Predicted to be active in nucleus.
		rs10873876	ST6GALNAC3	ST6GALNAC3 belongs to a family of sialyltransferases that transfer sialic acids from CMP-sialic acid to terminal positions of carbohydrate groups in glycoproteins and glycolipids. Associated with hepatocellular carcinoma and prostate cancer (PMIDs: 35321246, 29465788)
		rs9290663	KCNMB2	Encodes a protein important in regulating the flow of potassium ions across cell membranes and plays a role in various physiological processes, such as neuronal excitability, smooth muscle contraction, and hormone secretion. Associated with following cancers: non-small cell lung cancer, esophageal cancer, bladder cancer, cervical cancer, and endometrial cancer. (PMIDs: 35979065, 34516362, 34367236, 34026626, 33028109, 31539276)
		rs11155133	LOC102723724	RNA gene of ncRNA class
[23]	AML disease risk	rs7090018	FGFR2	Gene encodes a protein that engages with fibroblast growth factors, initiating a series of subsequent signals that ultimately impact cell division and differentiation. Associated with AML, T-cell ALL, mixed phenotype acute leukemia, and chronic myeloid leukemia. (PMIDs: 30668205, 36333298, 33049052, 31502137)
		rs2912759		
[24]	In vitro Ara-C chemosensitivity	rs1376041	ADGRG1	Receptor involved in cell adhesion and probably involved in cell–cell interactions. Mediates cell matrix adhesion in developing neurons and hematopoietic stem cells. Reported to play a role in leukemogenesis (PMID: 23478665) and is part of a 17-gene stemness score that aids in determining leukemia risk (PMID: 27926740)
		rs721947	93kb 3′ of NEDD1	Predicted to be involved in protein localization to centrosome. Associated with B-cell lymphoma, renal cell carcinoma, and cell cycle arrest. PMIDs: 36840486, 36347549, 23106787)
		rs75400242	IGF1R	Receptor binds insulin-like growth factor with a high affinity. Highly overexpressed in most malignant tissues, where it functions as an anti-apoptotic agent by enhancing cell survival. Associated with B-cell ALL, regulation of proliferation of AML stem cells, and T-cell ALL. (PMIDs: 37298628, 30013477, 27532210)
[25]	AML disease risk	rs75797233	BICRA	Enables transcription regulator activator activity. Involved in positive regulation of transcription, DNA-templated. Associated with colorectal cancer and lung cancer risk. (PMID: 30291333, 30128886)
[26]	AML and cytogenetically normal AML	rs4930561	KMT5B	This gene encodes a protein that contains a SET domain. SET domains appear to be protein–protein interaction domains that mediate interactions among a family of proteins that display similarity to dual-specificity phosphatases (dsPTPases). The function of this gene has not been determined. Associated with glioblastoma, sarcomas, and tumor recurrence. (PMIDs: 29967352, 36376321)
		rs3916765	LOC102725019	RNA gene of lncRNA class
[27]	De novo AML with and without abnormal cytogenetics, de novo MDS, and therapy-related AML and MDS	rs12203592	IRF4	Plays a role in regulation of interferons in response to infection by a virus and interferon-inducible genes. Lymphocyte specifically and negatively regulates Toll-like-receptor (TLR) signaling, which is important in the activation of innate and adaptive immune systems. Associated with B-cell malignancies, adult T-cell leukemia, childhood acute leukemias, and chronic myeloid leukemia (PMIDs: 36495369, 36446869, 34775495, 11013272)
